# Hospital days attributable to immune reconstitution inflammatory syndrome in persons living with HIV before and after the 2012 DHHS HIV guidelines

**DOI:** 10.1186/s12981-017-0152-0

**Published:** 2017-05-02

**Authors:** Peter Liu, Rebecca Dillingham, Kathleen McManus

**Affiliations:** 1 Department of Internal Medicine, University of Virginia, PO Box 801379, Charlottesville, VA 22908 USA; 2 Division of Infectious Diseases and International Health, University of Virginia, PO Box 801379, Charlottesville, VA 22908 USA

**Keywords:** Human immunodeficiency virus, HIV, Immune reconstitution inflammatory syndrome, IRIS, Hospitalization, Practice guidelines, United States Dept. of Health and Human Services, Acquired immunodeficiency syndrome, AIDS

## Abstract

**Background:**

Immune reconstitution inflammatory syndrome (IRIS) can manifest with initiation or reintroduction of antiretroviral therapy (ART) in persons living with HIV (PLWH). In 2012, updated United States treatment guidelines recommended initiation of ART for all PLWH regardless of CD4 count. The objectives of this study were to quantify hospital usage attributable to IRIS and assess the reasons for hospitalization in PLWH before and after the guideline update.

**Methods:**

Subjects were PLWH between 18–89 years of age who were hospitalized between November 1, 2009 and July 31, 2014. Equivalent time periods before and after updated treatment guidelines were considered, and designated as Time Period 1 and Time Period 2, respectively. IRIS-attributable hospitalizations were identified by ICD9 codes and electronic medical record searches with subsequent review and confirmation. For hospitalizations that were not confirmed as being IRIS-attributable, primary discharge diagnoses were reviewed.

**Results:**

A total of 278 PLWH were hospitalized 521 times throughout the study period. Time Period 1 had 9 PLWH with 12 IRIS-attributable hospitalizations while Time Period 2 had 6 PLWH with 9 IRIS-attributable hospitalizations. A larger proportion of IRIS-attributable hospital days was observed in Time Period 1 compared to Time Period 2 (7.5 vs 4.2%; p < 0.001). Median length of stay for IRIS-attributable hospitalizations was longer than for other diagnoses, particularly during Time Period 1 (12.0 vs 4.0; p = 0.05). The most common causes for hospitalizations in PLWH were non AIDS-defining infection, AIDS-defining malignancy, and gastrointestinal. PLWH who had HIV viral suppression (<200 copies/mL) accounted for 34 and 24% of hospitalizations in Time Periods 1 and 2 respectively.

**Conclusions:**

Hospitalizations for PLWH continue at high rates and IRIS is a significant contributing factor. In our single-center study, there was a lower number of IRIS-attributable hospitalizations and IRIS-attributable hospital days in Time Period 2 compared with Time Period 1. The hospital burden of IRIS may decrease over time as more PLWH are started on ART earlier in the course of infection. This study highlights the continued importance of early diagnosis and linkage to care of those infected with HIV, so that morbidity and costs associated with IRIS continue to decline.

## Background

Anti-retroviral therapy (ART) utilizes combination drug therapy to suppress replication of human immunodeficiency virus (HIV) and facilitates restoration of host immune function by increasing CD4 positive T-lymphocytes. The advent and wide usage of ART marked a paradigm shift in the management of persons living with HIV (PLWH) [1]. Multi-disciplinary efforts have yielded many novel agents that are both highly potent and generally well-tolerated making the use of ART more ubiquitous. As a result, the life expectancy for persons living with HIV (PLWH) has increased, and within this group, conditions experienced by the general population have become more prevalent [2–6].

While numerous clinical benefits are derived from ART, immune reconstitution inflammatory syndrome (IRIS) is a morbid complication that can manifest with initiation or reintroduction of ART. IRIS is broadly categorized into two pathophysiologic processes of ‘unmasking’ an untreated sub-clinical opportunistic infection (OI) or a ‘paradoxical worsening’ of a previously treated infection upon commencement of ART. Well described micro-organisms associated with IRIS include Mycobacterium tuberculosis, Cryptococcus, Cytomegalovirus (CMV), and Herpes virus, among others [7–16].

While the immuno-pathology of IRIS has yet to be completely defined, existing studies suggest that it is multifactorial and complex and includes elements of immune system dysregulation and restructuring of immune cell quantity and function [1, 7]. Several biomarkers have been identified as being disproportionately elevated in the setting of IRIS including TNF-α, IF-ɣ, and numerous interleukins [17–24]. Regulatory T- cells appear to demonstrate an impaired functional ability to abrogate over-exuberant immunological responses to antigenic stimuli despite an absolute quantitative expansion [25]. More recent studies also highlight the importance of innate immune system dysfunction with high pathogen burden in the development of IRIS [26, 27].

Clinicians are often confronted with the difficult task of discerning between IRIS and a more typical presentation of undertreated primary disease. The clinical manifestations and pathogenesis of IRIS are highly variable and related to the affected organ systems and causative pathogen [17–19, 22]. Further compounding the problem is the absence of clear diagnostic criteria for IRIS with the exception of pathogen-specific definitions that have been proposed for Mycobacterium tuberculosis and Cryptococcus IRIS [26]. Risk factors for IRIS must also be recognized. Numerous studies suggest that severe immunodeficiency and the presence of an OI at the time of ART initiation, particularly if accompanied by a high pathogen burden or disseminated disease, are important considerations [1, 7, 8, 11, 14, 15, 26]. The diagnostic complexity of IRIS in the face of the variable prevalence of underlying OIs have led to estimates of IRIS incidence ranging from 6–47% in PLWH [7, 28, 29].

In 2012, the United States Department of Health and Human Services (DHHS) published updated guidelines recommending the initiation of ART in all HIV infected individuals regardless of their CD4 count at the time of diagnosis [30]. While clinicians must still consider other factors including patient willingness and barriers to adherence, the anticipated population effect is that ART is being initiated earlier and often before severe immunodeficiency develops. To the best of our knowledge, the comparison of the burden of IRIS on the healthcare system as measured by hospital days related to IRIS before and after the 2012 HIV management guideline updates has not been investigated. In this study, we compare hospital days related to IRIS at our 604 bed tertiary-care hospital both before and after updated ART guidelines were published and describe the reasons for hospitalization in PLWH.

## Methods

The Clinical Data Repository (CDR), an electronic real-time database of protected health information was used to identify subjects at the University of Virginia Medical Center (UVaMC), a 604 bed tertiary care referral hospital. Cases included persons between 8–89 years of age who were coded by the International Classification of Diseases, Ninth Revision (ICD9) as having a diagnosis of HIV/AIDS or who had a reactive HIV screening assay and were hospitalized at UVaMC between November 1, 2009 and July 31, 2014. Cases with hospitalizations possibly attributable to IRIS were identified by (1) ICD9 code 995.90 and (2) by use of a comprehensive electronic medical record search for the terms “Immune Reconstitution Inflammatory Syndrome” or “IRIS” in hospital notes. By these mechanisms, hospitalizations identified as possibly being attributable to IRIS were then manually reviewed and confirmed independently by two Infectious Diseases physicians who have considerable experience in caring for PLWH. Cases that were not consistent with IRIS despite being coded by ICD9 were excluded.

Equivalent time periods of 28.5 months before (November 1, 2009–March 15, 2012, designated as Time Period 1) and after (March 16, 2012–July 31, 2014, designated as Time Period 2) publication of updated treatment guidelines were considered. Electronic medical records were reviewed for clinical and demographic characteristics, reason for hospitalization, and length of stay.

Clinical characteristics including CD4 cell count and HIV–RNA Viral Load were defined as the values obtained during the examined hospitalization or the values that were taken before the hospitalization. Hepatitis B (HBV) co-infection was defined as a positive HBV surface antigen, HBV IgM core antibody, or detectable HBV Viral Load anytime during or prior to the examined hospitalization. Hepatitis C (HCV) co-infection was defined as a positive HCV antibody or detectable HCV Viral Load anytime during or prior to the examined hospitalization. Chronic HBV or HCV infection was defined as persistent detection of HBV surface antigen, HBV Viral Load, or HCV Viral Load on at least two occasions with one instance occurring during hospitalization or at the time of the laboratory result chronologically closest to the start of hospitalization.

The primary outcome considered was hospital usage related to IRIS measured by IRIS-attributable hospital days and the percentage of total hospital days for PLWH that were attributable to IRIS. Comparisons were made between Time Period 1 and Time Period 2. Reasons for non IRIS-attributable hospitalizations were ascertained from documentation of the primary discharge diagnoses from discharge summaries. Primary discharge diagnoses were then systematically grouped by organ system and diagnosis category for analysis. Categorizing documented diagnoses of AIDS-defining infection or AIDS-defining malignancy was based on the comprehensive list issued by the Centers for Disease Control and Prevention [31].

Clinical information about IRIS-attributable hospitalizations including type of IRIS and causative pathogen was obtained. Paradoxical IRIS cases were defined as cases in which worsening of a previously recognized OI was observed in temporal relation to recent ART implementation. Unmasking IRIS cases were defined as cases in which new onset of symptoms compatible with a previously unrecognized OI were seen in temporal relation to recent ART implementation [7–16]. Due to low numbers and unavailable data, statistical analyses were not performed.

Chi square analyses were performed on categorical data to determine statistically significant differences between groups. Fisher’s Exact Test was utilized when a Chi square test had an expected value in any cell of less than five. Continuous data was analyzed by a t test to determine statistically significant differences between groups. When comparing medians, the Mann-Whitney U test was utilized. The significance threshold for all tests was set at .05.

## Results

A total of 278 subjects were hospitalized 521 times throughout Time Periods 1 and 2. The entire study population included 69% male, 53% black, and 44% white subjects with a mean age of 47.5 years. Across the time periods, at the time of hospitalization, more than 40% of subjects had CD4 cell counts less than 200, while 31% had CD4 cell counts between 200 and 500, and 25% had CD4 cell counts greater than 500. Approximately 55% of subjects had HIV viral loads less than 200 copies/mL with 46% under 20 copies/mL, while 37% had poor HIV viral suppression with viral loads greater than 1000 copies/mL. Overall in the hospitalized PLWH, the rates of Hepatitis B and Hepatitis C Co-infection were 12 and 18%.

During Time Period 1, there were 151 subjects with 259 non IRIS-attributable hospitalizations, and 9 subjects with 12 IRIS-attributable hospitalizations (Table 1). The majority of patients hospitalized during Time Period 1, for either IRIS-attributable or non IRIS-attributable causes, were male and identified as Black or Hispanic. There were no significant differences in age, race/ethnicity, and rates of Hepatitis B or Hepatitis C co-infection in subjects admitted with IRIS compared to those admitted for alternate causes (Table 1). A greater proportion of subjects with IRIS-attributable hospitalizations (56%) compared to non IRIS-attributable hospitalizations (32%) had poor virologic control with HIV viral loads greater than 1000 copies/mL), but this difference was not statistically significant (p = 0.54). Subjects with IRIS-attributable hospitalizations were more likely to have CD4 cell counts less than 200 compared to subjects with non IRIS-attributable hospitalizations, 89 vs 39% (p = 0.04).

**Table 1:** Hospitalized patient clinical and demographic characteristics by IRIS and study period

	Total study duration n (%)	Subjects with Time Period 1 non IRIS-attributable hospitalization n (%)	Subjects with Time Period 1 IRIS-attributable hospitalization n (%)	p value	Subjects with Time Period 2 non IRIS-attributable hospitalization n (%)	Subjects with Time Period 2 IRIS-attributable hospitalization n (%)	p value
Total subjects	278	151	9		112	6	
Mean age	47.5	47.7	42.8	0.17	47.7	47.3	0.94
Sex^a^				0.73			0.66
Male	192 (69)	105 (70)	7 (78)		75 (67)	5 (83)	
Female	86 (31)	46 (30)	2 (22)		37 (33)	1 (17)	
Race/ethnicity				0.08			0.008
White	121 (44)	61 (40)	2 (22)		58 (52)	0 (0)	
Black	147 (53)	84 (56)	5 (56)		53 (47)	5 (83)	
Hispanic	5 (2)	4 (3)	1 (11)		0 (0)	0 (0)	
Other/unknown	5 (2)	2 (1)	1(11)		1 (1)	1 (17)	
CD4 count^b^				0.004			0.18
<200	112 (40)	59 (39)	8 (89)		40 (36)	5 (83)	
200–500	86 (31)	47 (31)	1 (11)		37 (33)	1 (17)	
>500	69 (25)	39 (26)	0 (0)		30 (27)	0 (0)	
HIV viral load^c^				0.54			0.04
<200	154 (55)	89 (59)	4 (44)		60 (54)	1 (17)	
200–1000	10 (4)	7 (5)	0 (0)		2 (2)	1 (17)	
>1000	103 (37)	49 (32)	5 (56)		45 (40)	4 (66)	
Hepatitis B co-infection^d^				0.47			0.21
Chronic infection	59 (21)	15 (10)	2 (22)		15 (13)	1 (17)	
Exposure and clearance	33 (12)	35 (23)	1 (11)		20 (18)	3 (50)	
No exposure or infection	162 (58)	88 (58)	5 (56)		67 (60)	2 (33)	
Hepatitis C co-infection^e^				0.58			1.00
Chronic infection	16 (6)	31 (21)	1 (11)		18 (16)	1 (17)	
Exposure and clearance	51 (18)	10 (7)	1 (11)		5 (4)	0 (0)	
No exposure or infection	189 (68)	99 (66)	6 (67)		79 (71)	5 (83)	

^a^In our health system, subjects self-identify as male or female. This information is recorded in the electronic health record

^b^Time Period 1: 154 had a CD4 cell count measured, Time Period 2: 113 had a CD4 cell count measured

^c^Time Period 1: 154 had a HIV Viral Load measured, Time Period 2: 113 had a HIV Viral Load measured

^d^Time Period 1: 146 had HBV serologies measured, Time Period 2: 108 had HBV serologies measured

^e^Time Period 1: 148 had HCV serologies measured, Time Period 2: 108 had HCV serologies measured

Time Period 2 had 112 subjects with 241 non IRIS-attributable hospitalizations and 6 subjects with 9 IRIS-attributable hospitalizations. The majority of subjects hospitalized during Time Period 2 were male. Subjects who had IRIS-attributable hospitalizations were more likely to identify as Black compared to those with non IRIS-attributable hospitalizations, 83 vs 47% (p = 0.008). The age, race/ethnicity, and rates of Hepatitis B or Hepatitis C co-infections were similar for those with IRIS and non IRIS-attributable admissions. A greater proportion of subjects admitted with IRIS had poor virologic control with HIV viral loads greater than 1000 copies/mL (66 vs 40%, p = 0.04). During Time Period 2, patients with IRIS-attributable hospitalizations had more advanced immunodeficiency with 83% having CD4 cell counts less than 200 compared to 36% in those admitted for reasons unrelated to IRIS, although this difference was not statistically significant (Table 1).

**Fig. 1:**
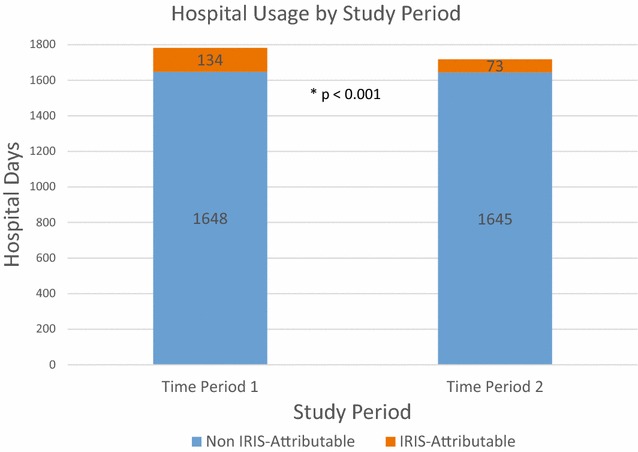
A comparison of hospital days for IRIS-attributable and non IRIS-attributable hospitalizations stratified by Study Time Period

There were more hospital days attributable to IRIS in Time Period 1 compared to Time Period 2, 134 and 73 days respectively, while the number of hospital days for non IRIS-attributable hospitalizations was stable (Fig. 1). There was a decrease in the number of all-cause hospitalizations from Time Period 1 to Time Period 2, but a larger proportion of hospital days were IRIS-attributable in Time Period 1 compared to the proportion in Time Period 2 (7.5 vs 4.2%, p < 0.001). The median length of stay for IRIS-attributable hospitalizations was longer than for other diagnoses in both Time Period 1 (12.0, interquartile range (IQR) 10 vs 4.0, IQR 5, p = 0.05) and Time Period 2 (5.5, IQR 8.25 vs 5.0, IQR 5.0, p = 0.58). The average length of stay for IRIS-attributable hospitalizations (11.1) was longer than for non IRIS-attributable hospitalizations (6.4) during Time Period 1. During Time Period 2, the average length of stay was 8.1 days for IRIS-attributable hospitalizations compared to 6.8 days for non IRIS-attributable hospitalizations.

Cases of paradoxical IRIS accounted for the majority of IRIS-attributable hospitalizations in both Time Period 1 (67%) and Time Period 2 (67%). Unmasking IRIS was observed in 33 and 22% of IRIS-attributable hospitalizations in Time Period 1 and Time Period 2 respectively. The most common causative pathogen of IRIS in Time Period 1 was Cryptococcus (42%), followed by Mycobacterium avium complex (17%), and JC Polyomavirus (17%). The most common causative pathogen of IRIS in Time Period 2 was Cryptococcus (22%), followed by Mycobacterium avium complex (22%) and Mycobacterium tuberculosis (11%). There was 1 case (8%) in Time Period 1 and 4 cases (44%) in Time Period 2 in which the causative pathogen was unknown. All cases of Cryptococcal IRIS (7, 100%) in our study manifest as paradoxical IRIS and 2 (50%) cases of MAC IRIS manifest as unmasking IRIS. During Time Period 1, 7 (58%) IRIS-attributable hospitalizations were for subjects on first-line ART, while 1 (8%) and 4 (33%) hospitalizations were for subjects on second-line and salvage ART respectively. During Time Period 2, 6 (67%) IRIS-attributable hospitalizations were for subjects on first-line ART, while 1 (11%) and 2 (22%) hospitalizations were for subjects on second-line and salvage ART respectively. Data regarding the exact duration of ART preceding IRIS-attributable hospitalizations and quantitative viral load trends were often unavailable due to prior care occurring at other institutions.

PLWH were hospitalized 271 times during Time Period 1 and 250 times during Time Period 2. IRIS-attributable hospitalizations accounted for 4.4% of hospitalizations in Time Period 1 and 3.6% of hospitalizations in Time Period 2. The most common cause for hospitalization in Time Period 1 was non-AIDS-defining infection accounting for 19.6% of hospitalizations. This was followed by gastrointestinal, AIDS-defining malignancy, and AIDS-defining infection. The most common cause for hospitalization in Time Period 2 was non-AIDS-defining infection accounting for 19.2% of hospitalizations. This was followed by AIDS-defining malignancy, gastrointestinal, and pulmonary (Tables 2, 3). During Time Period 1, 93 hospitalizations (34%) were for PLWH who had HIV Viral Loads less than 200 copies/mL, while 61 hospitalizations (24%) during Time Period 2 were for PLWH with similar viral suppression.

**Table 2:** Reason for hospitalization by organ system and diagnosis category

Diagnosis category	Number of admissions for PLWH in Time Period 1 (Nov. 1, 2009–Mar. 15, 2012) n (%)
Total admissions	271 (100)
1. Non-AIDS-defining infection	53 (19.6)
2. Gastrointestinal	30 (11.1)
3. AIDS-defining malignancy^a^	21 (7.7)
4. AIDS-defining infection^b^	17 (6.3)
5. Orthopedic	17 (6.3)
6. Pulmonary	16 (5.9)
7. Renal	13 (4.8)
8. Hematologic	13 (4.8)
9. Cardiovascular	12 (4.4)
10. IRIS	12 (4.4)
11. Psychological	12 (4.4)
12. Non-AIDS-defining malignancy	11 (4.1)
13. Neurological	8 (3.0)
14. Substance abuse	8 (3.0)
15. Symptom based	8 (3.0)
16. Other	8 (3.0)
17. Obstetric	7 (2.6)
18. Endocrine	5 (1.8)
19. Trauma	0 (0)

^a^AIDS Defining Malignancy category includes Invasive Cervical CA, Kaposi Sarcoma, Burkitt Lymphoma, Immunoblastic Lymphoma, Primary CNS Lymphoma

^b^AIDS Defining Infection category includes Candidiasis of the esophagus, bronchi, trachea, or lungs, invasive cervical cancer, disseminated or extrapulmonary Coccidioidomycosis, extrapulmonary Cryptococcosis, chronic Cryptosporidiosis, Cytomegalovirus disease (other than liver, spleen, or nodes), Cytomegalovirus retinitis, HIV related encephalopathy, chronic ulcers, bronchitis, pneumonitis, or esophagitis due to Herpes simplex, disseminated or extrapulmonary Histoplasmosis, chronic Isosporiasis, Kaposi’s Sarcoma, Burkitt’s Lymphoma, Immunoblastic Lymphoma, Primary CNS Lymphoma, disseminated or extrapulmonary disease due to non-tuberculous Mycobacterium, Mycobacterium tuberculosis of any site, Pneumocystis jirovecii pneumonia, recurrent pneumonia, Progressive Multifocal Leukoencephalopathy, recurrent Salmonella septicemia, Toxoplasmosis, HIV wasting syndrome [30]

**Table 3:** Reason for hospitalization by organ system and diagnosis category

Diagnosis category	Number of admissions for PLWH in Time Period 2 (Mar. 16, 2012–July 31, 2014) n (%)
Total admissions	250 (100)
1. Non-AIDS-defining infection	48 (19.2)
2. AIDS-defining malignancy^a^	35 (14.0)
3. Gastrointestinal	24 (9.6)
4. Pulmonary	24 (9.6)
5. AIDS-defining infection^b^	20 (8.0)
6. Cardiovascular	15 (6.0)
7. Neurological	13 (5.2)
8. Renal	12 (4.8)
9. Substance abuse	12 (4.8)
10. IRIS	9 (3.6)
11. Symptom based	8 (3.2)
12. Orthopedic	7 (2.8)
13. Non-AIDS-defining malignancy	7 (2.8)
14. Psychological	4 (1.6)
15. Obstetric	4 (1.6)
16. Hematologic	3 (1.2)
17. Other	3 (1.2)
18. Trauma	2 (0.8)
19. Endocrine	0 (0)

^a^AIDS Defining Malignancy category includes Invasive Cervical CA, Kaposi Sarcoma, Burkitt Lymphoma, Immunoblastic Lymphoma, Primary CNS Lymphoma

^b^AIDS Defining Infection category includes Candidiasis of the esophagus, bronchi, trachea, or lungs, invasive cervical cancer, disseminated or extrapulmonary Coccidiodomycosis, extrapulmonary Cryptococcosis, chronic Cryptosporidiosis, Cytomegalovirus disease (other than liver, spleen, or nodes), Cytomegalovirus retinitis, HIV related encephalopathy, chronic ulcers, bronchitis, pneumonitis, or esophagitis due to Herpes simplex, disseminated or extrapulmonary Histoplasmosis, chronic Isosporiasis, Kaposi’s Sarcoma, Burkitt’s Lymphoma, Immunoblastic Lymphoma, Primary CNS Lymphoma, disseminated or extrapulmonary disease due to non-tuberculous Mycobacterium, Mycobacterium tuberculosis of any site, Pneumocystis jirovecii pneumonia, recurrent pneumonia, Progressive Multifocal Leukoencephalopathy, recurrent Salmonella septicemia, Toxoplasmosis, HIV wasting syndrome [31]

## Discussion

In this retrospective study, there was a statistically significant decrease in hospital usage related to IRIS following the release of updated HIV treatment guidelines by the DHHS in 2012 with the proportion of hospital days attributable to IRIS in Time Period 2 being 4.2% compared to Time Period 1 of 7.5% (Chi square p < 0.001). The United States treatment guidelines issued by the DHHS prior to 2012 recommended ART for all patients with CD4 counts <350, however, the panel was divided on the strength of recommendation for patients with CD4 counts between 350 and 500, and half viewed ART as optional for patients with CD4 counts >500 [32]. With the 2012 guideline updates recommending the initiation of ART for PLWH regardless of the CD4 cell count at the time of diagnosis, it is plausible that a greater proportion of PLWH are being initiated on ART earlier in the course of HIV infection before progressive immunodeficiency develops and the likelihood of acquiring opportunistic infections rises.

The majority of subjects with an IRIS-attributable hospitalization in our cohort had CD4 cell counts less than 200 (mean 112) and detectable HIV Viral Loads in excess of 1000 copies/mL. This finding is similar to that of numerous prior studies which report on the association of severe immunodeficiency and the development of IRIS in PLWH [1, 7, 8, 26]. In a retrospective multicenter study evaluating the incidence and risk factors associated with Cryptococcal IRIS, Lortholary et al found that lower CD4 cell counts (<7 × 10^6^ cell/L, lower quartile) was an independent risk factor for the occurrence of IRIS [31]. In a separate study on M. *tuberculosis* IRIS in PLWH, Breton et al found that PLWH who developed IRIS had lower baseline CD4 cell counts (median 75), and that IRIS was associated with more pronounced reductions in HIV-1 RNA levels and increases in CD4 cell count percentages after 1 month of ART [34].

During both Time Period 1 and Time Period 2, subjects who identified as either Black or Hispanic were more likely to have IRIS compared to subjects who identified as White. Although findings have been heterogeneous, racial disparities, as it pertains to the care of PLWH and complications of HIV infection have been well described [35, 36]. In a multi-center analysis involving more than 10,000 subjects by Gebo et al, being of African American ethnicity was associated with a lower likelihood of receiving ART compared to Whites [37]. Similarly, in an analysis by Novak and colleagues, being of nonwhite race/ethnicity was significantly associated with the development of IRIS compared to non-Hispanic Whites (Adjusted OR: 1.50, p = 0.009) [28]. Reasons for these disparities are likely multi-factorial and include differences in access to care or insurance, variations in healthcare literacy, potential language barriers, stigma, and differences in rates of co-existing substance abuse [37–42].

The precise measurable impact of IRIS on PLWH and the healthcare system remain uncertain. Estimates on the incidence of IRIS range from 6–47% related to variable definitions of IRIS and the diversity of settings in which studies take place with different resources and prevalence of OIs [7, 28, 29, 32–34]. Yet while the majority of IRIS events are self-limiting, fatalities, particularly when pathogen specific IRIS involves the central nervous system are well documented [7, 22, 28, 33, 43]. Moreover, the morbidity and use of healthcare resources associated with IRIS are significant.

The median length of stay for subjects with IRIS-attributable hospitalizations was 8 days longer than for non IRIS-attributable hospitalizations during Time Period 1. The reasons for this are likely multiple. First, IRIS is a diagnostically complex entity without clear pathognomonic features, and exclusion of progression or inadequate treatment of an underlying primary OI is required. Second, the management of IRIS events are not standardized. Finally, PLWH who experience IRIS events often comprise a population with higher acuity of illness owing to associations with poorer baseline immunologic parameters and virologic control.

During both Time Period 1 and Time Period 2, Cryptococcus species and Mycobacterium avium complex (MAC) were the most frequent causes of IRIS-attributable hospitalizations. Cryptococcal IRIS was associated with paradoxical IRIS, and MAC with unmasking IRIS, although the latter was not exclusively observed. The relatively high rate of paradoxical Cryptococcal IRIS may be due to several factors. The incidence of paradoxical Cryptococcal IRIS is relatively high and has been reported at frequencies of 10–42% in ART naïve PLWH [23, 44, 45]. Compared to other etiologies of IRIS which can be self-limiting, Cryptococcal meningitis IRIS carries a high rate of morbidity and mortality which may necessitate hospital admission [45]. Moreover, prolonged or recrudescence of symptoms, which is not unusual for Cryptococcal IRIS, led to recurrent admissions in our study. One patient with paradoxical Cryptococcal IRIS accounted for 3 IRIS-attributable hospitalizations in Time Period 1, while a different patient accounted for 2 IRIS-attributable hospitalizations in Time Period 2.

With widely expanding usage of ART and the ease of several once daily regimens now available as first-line treatments, PLWH are living longer, but hospitalizations for PLWH continue at rates higher than the general population [46]. Over the course of our 57 month study period, PLWH were hospitalized 521 times with IRIS accounting for 4.4% of hospitalizations in the pre-guideline period and 3.6% of hospitalizations in the post-guideline period (Tables 2, 3). Importantly, 34% of hospitalizations in Time Period 1 and 24% of hospitalizations in Time Period 2 were for PLWH who demonstrated HIV viral suppression with HIV Viral Loads quantitated as undetectable or less than 200 copies/mL. This parallels findings from other studies that show higher rates of hospitalization in PLWH compared to the general population, even amongst those with viral suppression [46, 47].

The leading reason for hospitalization in both study periods was non-AIDS-defining infection accounting for greater than 19% of hospitalizations throughout. This is similar to findings in several other studies reporting on the causes for hospitalization in PLWH [46, 47]. Following non-AIDS-defining infection, gastrointestinal reasons represented the second most common cause of hospitalization in Time Period 1 (11.1%), and the third most common cause in Time Period 2 (9.6%). Gastrointestinal causes, particularly pancreatitis and liver disease have also been found to be common causes for admission in other studies examining hospitalization trends among PLWH [46, 47]. Co-existing substance abuse and hepatitis co-infection, which remains prevalent among PLWH, may be at least in part responsible for this trend.

AIDS-defining malignancy comprised a significant proportion of hospitalizations for PLWH in both the pre-guideline and post-guideline period accounting for 7.7 and 14.0% of admissions respectively. This is in contrast to lower rates that are reported in other studies including a large 8 year analysis of a United States military cohort in which hospitalizations for AIDS-defining malignancy was observed at a rate of only 2.3 per 1000 person-years [46]. While the high proportion of admissions related to AIDS-defining malignancy we observed is surprising, the majority of these hospitalizations were for a minority of the study population. In Time Period 1, 5 subjects accounted for the 21 hospitalizations for AIDS-defining malignancy, and in Time Period 2, 9 subjects accounted for 35 such hospitalizations. Moreover, the majority of these hospitalizations were for pre-scheduled chemotherapeutic admissions.

Limitations of this study include the retrospective study design and reliance on the accuracy of medical record documentation. Without universally accepted definitions for IRIS, we, like others, have relied on expert opinion for diagnostic purposes. IRIS events requiring inpatient care are an uncommon occurrence, and as such, the number of cases in our study was relatively low. Nevertheless, we did observe a reduction in the burden of IRIS on the healthcare system in the Time Period 2 compared to Time Period 1, and our study adds to the literature as the first to examine such a trend. While guideline updates were undoubtedly significant, other factors that were not evaluated in our study may have influenced our findings, such as rates of retention in care, rates of ART compliance, and growth of expertise with recognition of IRIS. Finally, PLWH who experienced IRIS events that were managed in the outpatient setting and who may have required significant healthcare resource allocation, were not captured in our study.

Future multi-center studies could elucidate whether fewer IRIS events requiring hospitalization is occurring on a broader scale. Further evaluation of the demographic characteristics, immunologic recovery and viral suppression trends, and ART regimens that are associated with an IRIS event would be informative. Continued efforts to clarify the complex pathogenesis of IRIS will be instructive as more standardized definitions of IRIS are developed.

## Conclusions

Hospitalizations continue at high rates for PLWH compared to the general population, and IRIS is an important contributing factor [46–50]. In our single center study, we observed a decrease in the burden of IRIS on the healthcare system as measured by a reduction in the percentage of IRIS-attributable hospital days in Time Period 2 compared with Time Period 1. As a greater proportion of PLWH are started on ART earlier in the course of HIV infection, immunologic control and suppression of viral replication may more readily occur prior to acquisition of OIs thereby reducing the risk of IRIS. Efforts to recognize and diminish healthcare disparities in HIV care must continue, so that individual and population-level benefits derived from early ART can be achieved across all groups.

## Abbreviations

IRIS: immune reconstitution inflammatory syndrome
ART: antiretroviral therapy
PLWH: persons living with HIV
HIV: human immunodeficiency virus
OI: opportunistic infection
CMV: cytomegalovirus
DHHS: United States Department of Health and Human Services
CDR: Clinical Data Repository
UVaMC: University of Virginia Medical Center
ICD9: International Classification of Diseases, Ninth Revision
HBV: Hepatitis B
HCV: Hepatitis C
IQR: interquartile range
MAC: mycobacterium avium complex
