# Erratum to: Hospital days attributable to immune reconstitution inflammatory syndrome in persons living with HIV before and after the 2012 DHHS HIV guidelines

**DOI:** 10.1186/s12981-017-0155-x

**Published:** 2017-05-22

**Authors:** Peter Liu, Rebecca Dillingham, Kathleen A. McManus

**Affiliations:** 1 Department of Internal Medicine, University of Virginia, PO Box 801379, Charlottesville, VA 22908 USA; 2 Division of Infectious Diseases and International Health, University of Virginia, PO Box 801379, Charlottesville, VA 22908 USA

## Erratum to: AIDS Res Ther (2017) 14:25 DOI 10.1186/s12981-017-0152-0

Following publication of this article [1], it has come to our attention that one of the authors should have had their middle initial included, Kathleen A. McManus. The original article has been updated to reflect this.

Furthermore, it was reported in the Methods section that the ages of patients involved in this study were “8–89” when in fact the correct range was 18–89. The original version of the article has been revised to reflect this.
